# *Vibrio tapetis*, the Causative Agent of Brown Ring Disease, Forms Biofilms with Spherical Components

**DOI:** 10.3389/fmicb.2015.01384

**Published:** 2015-12-08

**Authors:** Sophie Rodrigues, Christine Paillard, Gaël Le Pennec, Alain Dufour, Alexis Bazire

**Affiliations:** ^1^Université de Bretagne-Sud, EA 3884, LBCM, IUEMLorient, France; ^2^UMR 6539 Laboratoire des Sciences de l'Environnement Marin, Centre National de la Recherche Scientifique, Institut Universitaire Européen de la Mer, Université de Brest, UBO, IRD, IfremerPlouzané, France

**Keywords:** *Vibrio tapetis*, biofilm, Brown Ring Disease, *Ruditapes philippinarum*, spherical components, temperature effect, salinity effect

## Abstract

*Vibrio tapetis* is a marine bacterium causing Brown Ring Disease (BRD) in the Manila clam *Ruditapes philippinarum. V. tapetis* biofilm formation remains unexplored depite the fact that it might be linked to pathogenicity. Our objectives were to characterize the *in vitro* biofilm formation of *V. tapetis* and evaluate the effects of culture conditions. Biofilm structure and its matrix composition were examined by confocal laser scanning microscopy and scanning electron microscopy. *V. tapetis* was able to form biofilms on a glass substratum within 24 h. Polysaccharides and extracellular DNA of the biofilm matrixes were differently distributed depending on the *V. tapetis* strains. Spherical components of about 1–2 μm diameter were found at the biofilm surface. They contain DNA, proteins, and seemed to be physically linked to bacteria and of cellular nature. Transmission electron microscopy showed that the spherical components were devoid of internal compartments. Temperatures >21°C inhibit BRD whereas low salinity (2%) favor it, none of the both conditions altered *V. tapetis'* ability to form biofilms *in vitro*. We suggest therefore that biofilm formation could play a role in the persistence of the pathogen in clam than in BRD symptoms.

## Introduction

Bacteria belonging to *Vibrio* species are ubiquitous in aquatic ecosystems and can have symbiotic or pathogenic relationships with eukaryotic hosts. They can cause vibrioses in various marine organisms, including molluscs (Paillard et al., 2004), and many diseases affecting shellfishes have been reported (Pass et al., 1987; Friedman et al., 1991; Le Roux et al., 2002). *Vibrio tapetis* is the causative agent of clams' infection called Brown Ring Disease (BRD; Paillard et al., 1989; Paillard and Maes, 1990; Borrego et al., 1996a). BRD is a chronic shell disease characterized by a brown deposit of conchiolin onto the inner edge of the clam shell. This deposit formation is induced by *V. tapetis* attachment and colonization of periostracal lamina and shell secretions of the clam (Paillard and Maes, 1995b). The attachment of *V. tapetis* to the periostracal lamina is the initial event in the BRD process. Colonization by *V. tapetis* provokes some alterations and ruptures the periostracal lamina, allowing the bacterial penetration into the extrapallial fluids where they are progressively embedded into the conchiolin deposit layers. If some tissue lesions occur, *V. tapetis* can penetrate and proliferate into tissues, which can lead to the clam death (Paillard, 1992, 2004a,b; Allam et al., 1996, 2000, 2002).

In general, one key factor for environmental survival and dissemination of bacteria and for host infection is the ability to form biofilms (Costerton et al., 1999). Biofilms are composed of cells bound to a surface and to each other and embedded within a matrix of extracellular polymeric substances that they have produced (Donlan, 2002). Biofilms enhance bacterial growth and survival by providing access to nutrients and provide protection from predators and antimicrobial compounds. Furthermore, pathogenicity is often closely related to bacterial biofilm formation (Parsek and Singh, 2003; Huq et al., 2008). Several studies investigated the biofilm formation by *Vibrio* species (Yildiz and Visick, 2009; Vezzulli et al., 2015). However, most of the information about *Vibrio* biofilms is coming from studies of human pathogenic species, in particular the etiological agent of cholera, *Vibrio cholerae*.

Studies on *V. tapetis* pathogenicity have shown that the clam periostracum colonization by *V. tapetis* is a required step in its infectious cycle (Paillard and Maes, 1995a,b; Borrego et al., 1996b; Lopez-Cortes et al., 1999). This bacterial attachment onto the periostracum suggests that biofilm is subsequently developed. This notion is supported by a Brown Ring syndrome microscopic study, which revealed bacterial aggregates within the conchiolin deposit (Paillard and Maes, 1995b). Whereas BRD development was the object of a number of previous studies (Paillard, 1992, 2004a,b; Allam et al., 1996, 2000, 2002), no study describing the *V. tapetis* biofilms have been led so far. More generally, data on biofilm formation by *Vibrio* pathogens of marine organisms are extremely scarce. In the present work, we evaluated biofilm formation by *V. tapetis* and characterized it, from the description of first steps of biofilm formation to the extracellular matrix. Unexpectedly, we observed spherical components which we describe here. Moreover, the effects of culture conditions on biofilm development were examined.

## Materials and methods

### Bacterial strains and culture conditions

The bacterial strains used in this study are listed in Table 1. The CECT4600 and IS1 strains were isolated from the venerid clam *R. philippinarum* suffering from BRD (Paillard and Maes, 1990). Strains isolated from other species of venerid clams harboring BRD symptoms, such as *V. tapetis* GDE and GTR I (isolated from *Dosinia exoleta* and *Tapes rhomboides*, respectively), have been identified as *V. tapetis* using Multi Locus Sequence Analyses (*gyrB, recA, rpoA*, and *pyrH* genes) and/or DNA-DNA hybridization methods (P. Le Chevalier, C. Paillard et al., unpublished data). The *V. tapetis* strains CECT4600 [wild type and its derivative producing the Green Fluorescent Protein (GFP)] and IS1 induce BRD by pallial infection of *R. philippinarum* (Paillard and Maes, 1990 and C. Paillard, unpublished data). In contrast, the strains LP2 (isolated from the wrasse fish *Symphodus melops*) and GDE are not able to reproduce BRD after pallial inoculation in the clam *R. philippinarum* (Paillard, 2004b; Trinkler, 2009). The strain GTR I, isolated from Brown ring diseased *Tapes rhomboïdes*, is able to induce BRD in this species (Le chevalier and Paillard, unpublished data). All *V. tapetis* strains were stored at −80°C in 25% glycerol and were routinely grown aerobically in Zobell medium [g.l^−1^: tryptone, 4; yeast extract, 1; sea salts (Sigma), 30; ferric phosphate, 0.1] at 18°C, the optimal temperature for *V. tapetis* growth. Kanamycin (Km) was used at 100 μg.ml^−1^ during precultures of *V. tapetis* CECT4600-GFP but not during biofilm culture in order to grow the biofilms of this strain in the same conditions as those of *V. tapetis* CECT4600 without plasmid and *V. tapetis* LP2, which are Km-sensitive. The growth rates were determined from growth kinetics recorded for each strain at 18°C in Zobell medium at 18°C in a 96-well microplate. OD_600_ measurements were made every 15 min for 48 h using a plate reader (Bioscreen C, Oy Growth Curves Ab Ltd, Finland). Planktonic cells grown at 18°C in Zobell medium during 24 or 48 h were observed with DM6000B microscope (Leica Microsystems, Heidelberg, Germany), using a 63x oil immersion objective.

**Table 1:** *****V. tapetis*** strains used in this study**.

**Strain**	**Characteristics**	**Growth rate (μ)**	**Host**	**Geographic origin**	**References**
CECT4600	Wild type strain,	0.32 h^−1^	Venerid clam, *R. philippinarum*	Landeda, France	Paillard and Maes, 1990
CECT4600-GFP	CECT4600 containing the pVSV102 plasmid (Km^R^, *gfp*)	0.32 h^−1^	Venerid clam, *R. philippinarum*		Travers et al., 2008, LEMAR collection^a^
LP2	Wild type strain	0.38 h^−1^	Wrasse fish *Symphodus melops*	Bergen, Norway	Jensen et al., 2003
IS1 (VP1)	Wild type strain	0.34 h^−1^	Venerid clam, *R. philippinarum*	Landeda, France	Paillard and Maes, 1990
GDE	Wild type strain	0.34 h^−1^	Venerid clam *Dosinia exoleta*	Glenan island, France	LEMAR collection^a^
GTR I	Wild type strain	0.35 h^−1^	Venerid clam *Tapes rhomboides*	Glenan island, France	LEMAR collection^a^

a *Bacterial collection of the LEMAR laboratory: Université de Brest, CNRS, IRD, Ifremer, UMR 6539 Laboratoire des Sciences de l'Environnement Marin, Institut Universitaire Européen de la Mer, Technopôle Brest Iroise, Plouzané, France*.

### Biofilm culture

*V. tapetis* biofilms were grown at 18°C under hydrodynamic conditions in a three channel flow cell (1 × 4 × 44 mm; Biocentrum, DTU, Denmark; Pamp et al., 2009). The flow system was assembled, prepared, and sterilized as described by Tolker-Nielsen and Sternberg (2011). The substratum consisted of a microscope glass coverslip [24 × 50 st1 (KnittelGlasser, Braunschweig, Germany)]. Each channel was inoculated with 250 μl of an overnight culture of *V. tapetis* diluted to an OD_600_ of 0.1 in Artificial Sea Water [ASW: 30 g.l^−1^ sea salts (Sigma Aldrich, Saint-Louis, MO)]. A 2-h attachment step was performed without any flow of ASW or medium. A flow (2.5 ml.h^−1^) of Zobell medium was then applied for 24 to 48 h using a Watson Marlow 205U peristaltic pump (Watson Marlow, Falmouth, UK). For experiments addressing the effects of higher osmotic conditions or temperatures, a Zobell medium with 50 g.l^−1^ of sea salts (Zobell 5%) was used or the temperature was set to 23°C during biofilm culture, respectively. The biofilms were then either observed by confocal laser scanning microscopy (CLSM) or by electron microscopy (SEM), as described below.

### Confocal laser scanning microscopy (CLSM)

Biofilms formed by *V. tapetis* CECT4600-GFP (Table 1) were observed by monitoring the GFP fluorescence. Depending on the biofilm constituents to observe, various fluorescent dyes were used after biofilm growth. The non GFP-producer bacteria were routinely stained with 5 μM SYTO 9 (Invitrogen, Carlsbad, Ca). 0.3 μM propidium iodide (PI; Invitrogen, Carlsbad, Ca) was used to examine the permeability of spherical components. Additional dyes were used to detect matrix components: polysaccharides and extracellular DNA (eDNA) were stained with 100 μM Calcofluor White (Sigma Aldrich, Saint-Louis, Mo; Chen et al., 2007) and 1 μM 7-hydroxy-9H-(1,3-dichloro-9,9-dimethylacridin-2-one, DDAO) (Invitrogen, Carlsbad, Ca; Allesen-Holm et al., 2006), respectively. All stainings were performed after biofilm growth by injecting 250 μl of the appropriate fluorescent dye(s) prepared in ASW into a flow cell channel, incubating at room temperature for 15 min in the dark, and washing for 15 min with a flow (2.5 ml.h^−1^) of Zobell medium. CLSM observations were then immediately performed with a TCS-SP2 microscope (Leica Microsystems, Heidelberg, Germany), using a 63x oil immersion objective. GFP and SYTO 9 were excited at 488 nm and fluorescence emission was detected between 500 and 550 nm. PI and DDAO were excited at 543 and 633 nm, respectively, and fluorescence emissions were detected at 565 and 660 nm, respectively. Calcofluor White was excited at 400 nm and fluorescence emission was detected between 410 and 450 nm. Fluorescence signals of double-labeled or triple-labeled specimens were acquired simultaneously. Images were taken every micrometer throughout the whole biofilm depth. For visualization and processing of three-dimensional (3D) image data (volume rendering with shadow projection), the Leica LAS AF software (Leica Microsystems, Heidelberg, Germany) was used. Quantitative analyses of image stacks were performed using the COMSTAT software (http://www.imageanalysis.dk/; Heydorn et al., 2000). At least three image stacks from each of three independent experiments (nine stacks in total) were used for each analysis.

### Scanning electron microscopy (SEM)

Fragments of flow cell glass substratum, on which a 48 h biofilm was formed, were aseptically retrieved and immerged overnight in a 2.5% glutaraldehyde solution at 4°C for biological material fixation. Samples were then washed three times in 0.1 M marine phosphate buffer (pH 7.35) for 10 min at 20°C and gradually dehydrated in an ethanol series (50, 70, 95, and 100%, 3 times for 15 min each). The samples were dried in ethanol in a CPD 030 critical point dryer (Bal-Tec, Bondoufle, France), using CO_2_ as a transitional fluid until the critical point was reached. The samples were mounted on aluminum stubs and coated for 120 s at 20 mA with gold-palladium alloy, using a model 501 sputter coater (Edwards Pirani, West Sussex, United Kingdom), and were observed with a JEOL 6460LV microscope (JEOL Ltd. Tokyo, Japan) at magnifications of × 1000 to × 20,000.

### Transmission electron microscopy (TEM)

To observe thin sections of bacterial cells from biofilms, the glass slide was broken and cells detached from the biofilm but still present in the flow cell channel were harvested by pipetting and pelleted by centrifugation at 4000 × g for 10 min. The samples were fixed overnight in a 2.5% glutaraldehyde solution at 4°C and washed twice with a solution of sodium cacodylate 0.1 M (prepared in ASW) for 30 min. Samples were placed for 1 h in a post-fixation solution of 1% osmium tetroxide (prepared in sodium cacodylate 0.1 M, pH 7.35, 1100 milliosmoles). They were gradually dehydrated in a graded series of ethanol (70, 95, and 100% twice for 30 min each) and infiltrated with an ascending series of Spurr's low-viscosity embedding medium in ethanol (Electron Microscopy Sciences, Hatfield, PA; 25, 50, 75, and 100%) and cured at 70°C for 8 h. Ultrathin sections were cut at 60 nm with a Leica Reichert Ultracut S (Leica Microsystems, Heidelberg, Germany) and collected on 300 mesh square Cu/Rh grids (EMS, Hatfield, USA). Sections were stained with uranyl acetate and lead citrate prior to examination using a JEOL JEM 1400 transmission electron microscope (JEOL Ltd, Tokyo, Japan).

## Results

### Biofilm structure and formation

**Figure 1:**
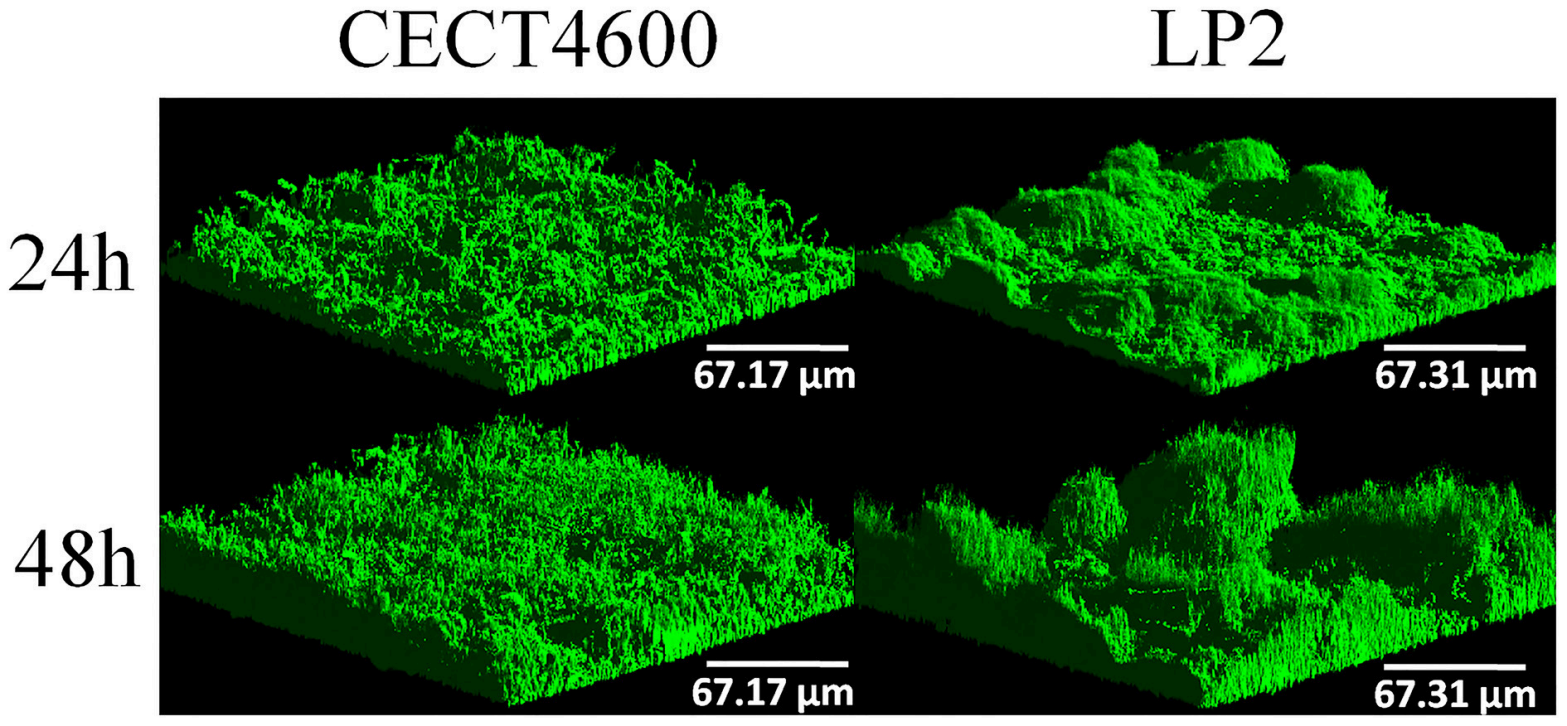
**Three-dimensional (3D) views of ***V. tapetis*** CECT4600 and LP2 biofilms grown for 24 and 48 h**. Bacteria were stained with SYTO 9 and observed by CLSM.

**Figure 2:**
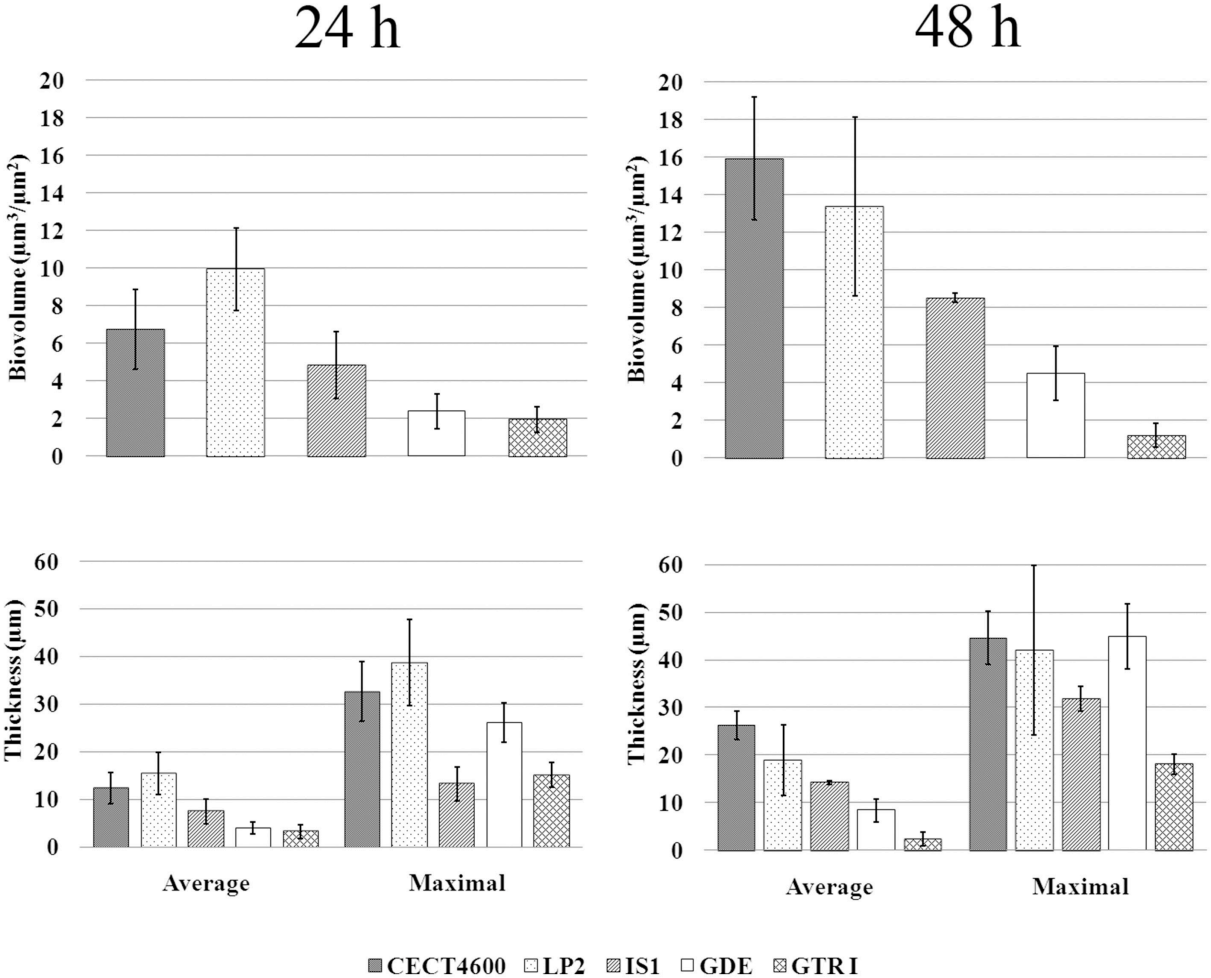
**Biovolume and thickness values of biofilms of the indicated ***V. tapetis*** strains**. The values were determined by image analyses using the COMSTAT software. Data are the means for at least three observations from three independent experiments (at least nine results), standard deviations are shown as a bar. Average biovolume of 24 and 48 h biofilms are significantly different (*p* < 0.001 for each strain except for GTR1, *p* < 0.05).

We first compared biofilm formation by two *V. tapetis* strains: CECT4600, which is the type strain (Borrego et al., 1996a) and is responsible for BRD in *R. philippinarum* (Paillard and Maes, 1990); and LP2 (Jensen et al., 2003), which is not able to enhance BRD in *R. philippinarum* after pallial inoculation (Choquet, 2004; Paillard, 2004b), but is a pathogen of the wrasse fish *Symphodus melops* (Table 1). Both strains displayed similar growth rates in liquid cultures (Table 1) and were able to form biofilms on glass in our culture conditions. *V. tapetis* CECT4600 formed non-structured and rather homogeneous biofilms (Figure 1) reaching an average thickness of 26 μm and a biovolume of 16 μm^3^.μm^−2^ after 48 h of growth (Figure 2). These two parameters increased about 2-fold between 24 and 48 h of growth (Figure 2). *V. tapetis* LP2 biofilms were visually very different since they displayed a structured architecture with mushroom-like components (Figure 1). The average thickness and biovolume were higher at 48 h (19 μm and 14 μm^3^.μm^−2^, respectively) than at 24 h, but they increased only by factors 1.2–1.4 between these two time points (Figure 2). A time-lapse study using light microscopy in which an image was recorded every 10 min during the first 18 h of biofilm growth revealed that the CECT4600 cells attached to the glass surface and colonized the whole surface by dividing, but did not display a surface motility behavior such as swarming or twitching (see Supplemental Movie 1). Cell multiplication started 2 h after the attachment step. Between 2 and 8 h post-attachment, bacteria were multiplying, yielding cell aggregates. From 8 h, the first microcolonies were observed, and the surface was completely covered by cells after 12 h. Similar observations were made for the LP2 strain, except that the building of mushroom-like structures was apparent after 10 h, and detachment of large biofilm parts was clearly observed from 13 h, which could contribute to the shaping of 3D structures (see Supplemental Movie 2).

### Distribution of matrix components

**Figure 3:**
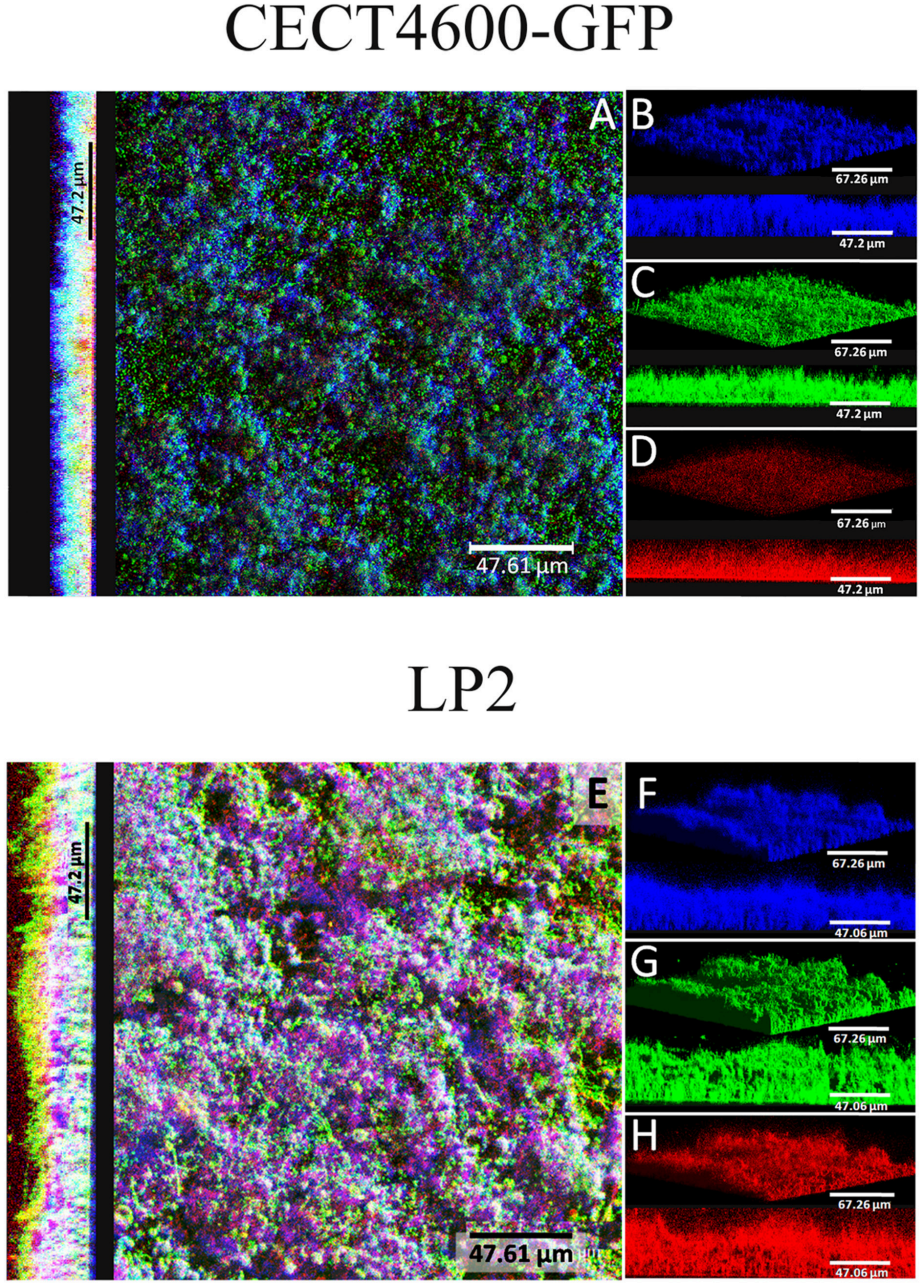
**Extracellular matrix compounds of 24 h ***V. tapetis*** CECT4600-GFP and LP2 biofilms stained by Calcofluor White (β1-3 and β1-4 polysaccharides) and DDAO (eDNA)**. **(A,E)**: top views and side views of overlay; **(B–D)** and **(F–H)**: 3D views and side views of β-polysaccharides (stained in blue), bacteria (stained in green) detected by GFP fluorescence (CECT4600-GFP) or SYTO 9 staining (LP2), or eDNA (stained in red) staining.

**Figure 4:**
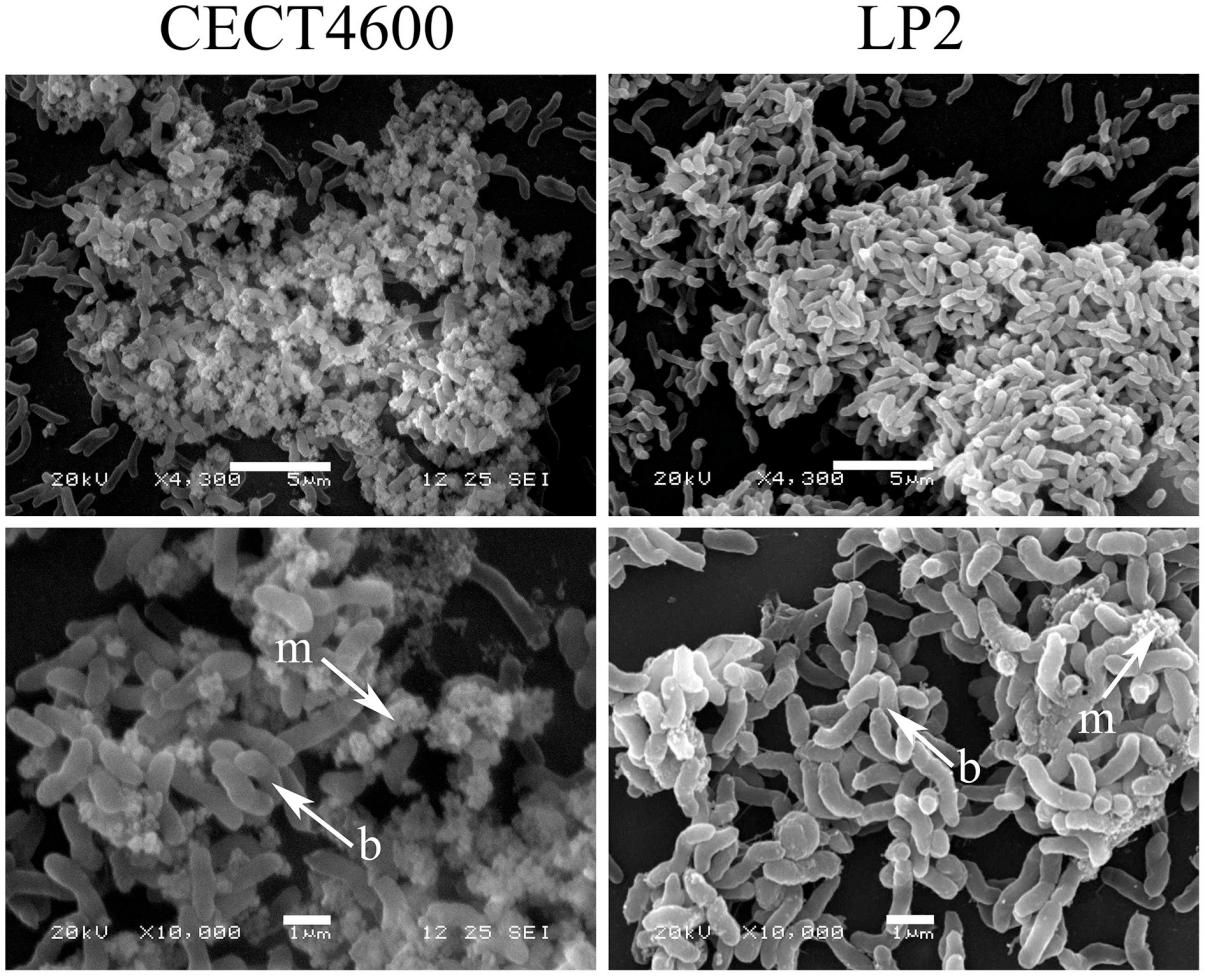
**SEM images of 48 h biofilms formed by ***V. tapetis*** CECT4600 and LP2**. Arrows indicates bacterial cells (b) and extracellular matrix (m). Scale bars: 5 μm (top panels); 1 μm (bottom panels).

Specific fluorescent dyes allowed the CLSM observation of matrix components and of their distribution within biofilms. To facilitate multi-labeled observations, we used the CECT4600-GFP strain (Table 1) after verifying that the presence of the GFP-encoding plasmid pVSV102 (Travers et al., 2008) did not affect the biofilm architecture (Figure 3C) and the biofilm parameters (biovolume, and average and maximal thicknesses; data not shown). The composition of the extracellular matrix was examined by staining the biofilms with Calcofluor White (binds to β1-3 and β1-4 polysaccharides) and DDAO (binds to eDNA). Both dyes were able to stain the matrixes of CECT4600-GFP and LP2 biofilms. Extracellular DNA was not visible on the top view of CECT4600-GFP biofilms (Figure 3A, top view), but was located at the biofilm base, roughly at its lowest 1/3 part (Figure 3A, side view, and Figure 3D). By contrast, eDNA was observed throughout the LP2 biofilms (Figures 3E,H). CECT4600-GFP biofilms displayed a homogeneous distribution of polysaccharides (Figure 3B), which were clearly visible at the top of the biofilms (Figure 3A). In LP2 biofilms, the polysaccharides were mostly found in the lowest 3/4 part of the biofilm (Figure 3E, side view, and Figure 3F) and were not predominant at the biofilm surface (Figure 3E, top view). When using SEM to observe the biofilm surfaces, the matrix was clearly visible at the top of CECT4600-GFP biofilms, but much less abundant on LP2 biofilms (Figure 4). The CLSM data (Figure 3) indicate that the matrix observed by SEM is mainly constituted of polysaccharides.

### *V. tapetis* biofilms display unusual components

**Figure 5:**
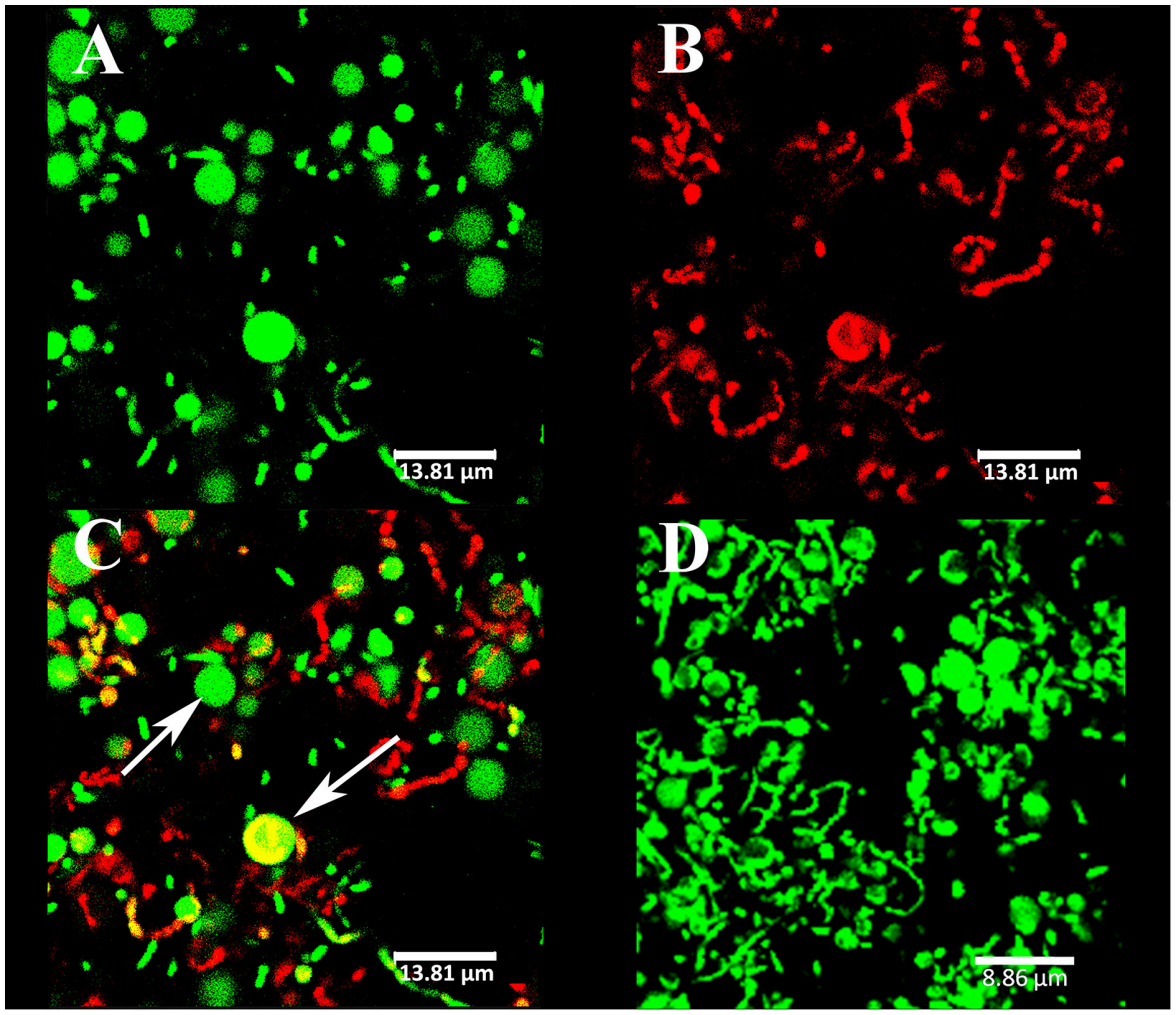
**Spherical components observed at the biofilm surface by CLSM**. The strains were *V. tapetis* CECT4600-GFP **(A–C)** and CECT4600 **(D)**. GFP fluorescence was detected **(A)** or the following DNA-binding dyes were used: propidium iodide **(B)**, same biofilm part as in **(A)** or SYTO 9 **(D)**. **(C)**: overlay of GFP and propidium iodide fluorescences from **(A,B)**. The arrows indicate spherical components.

CLSM observations revealed the presence of spherical components at the surface of CECT4600-GFP biofilms (Figures 5A–C) and of SYTO 9-stained CECT4600 biofilms (Figure 5D). The fact that these components displayed a green fluorescence when the bacteria carried the GFP-encoding plasmid pVSV102 (Figure 5A) revealed that they contain GFP, which suggests that they are likely to contain other proteins synthesized by the bacteria. Furthermore, the spherical components could be stained with the DNA-binding dyes propidium iodide (PI) (Figure 5B) and SYTO 9 (Figure 5D), indicating that they should contain DNA. The overlay of GFP and PI fluorescence revealed that only few spherical components were labeled by PI (Figure 5C), suggesting that only damaged spherical components were permeable to PI. We failed to detect these components in 24 and 48 h liquid cultures in Zobell medium at 18°C of *V. tapetis* CECT4600 grown without Km and CECT4600-GFP grown in the presence or not of Km (data not shown), they therefore seem to be biofilm-specific in our culture conditions.

**Figure 6:**
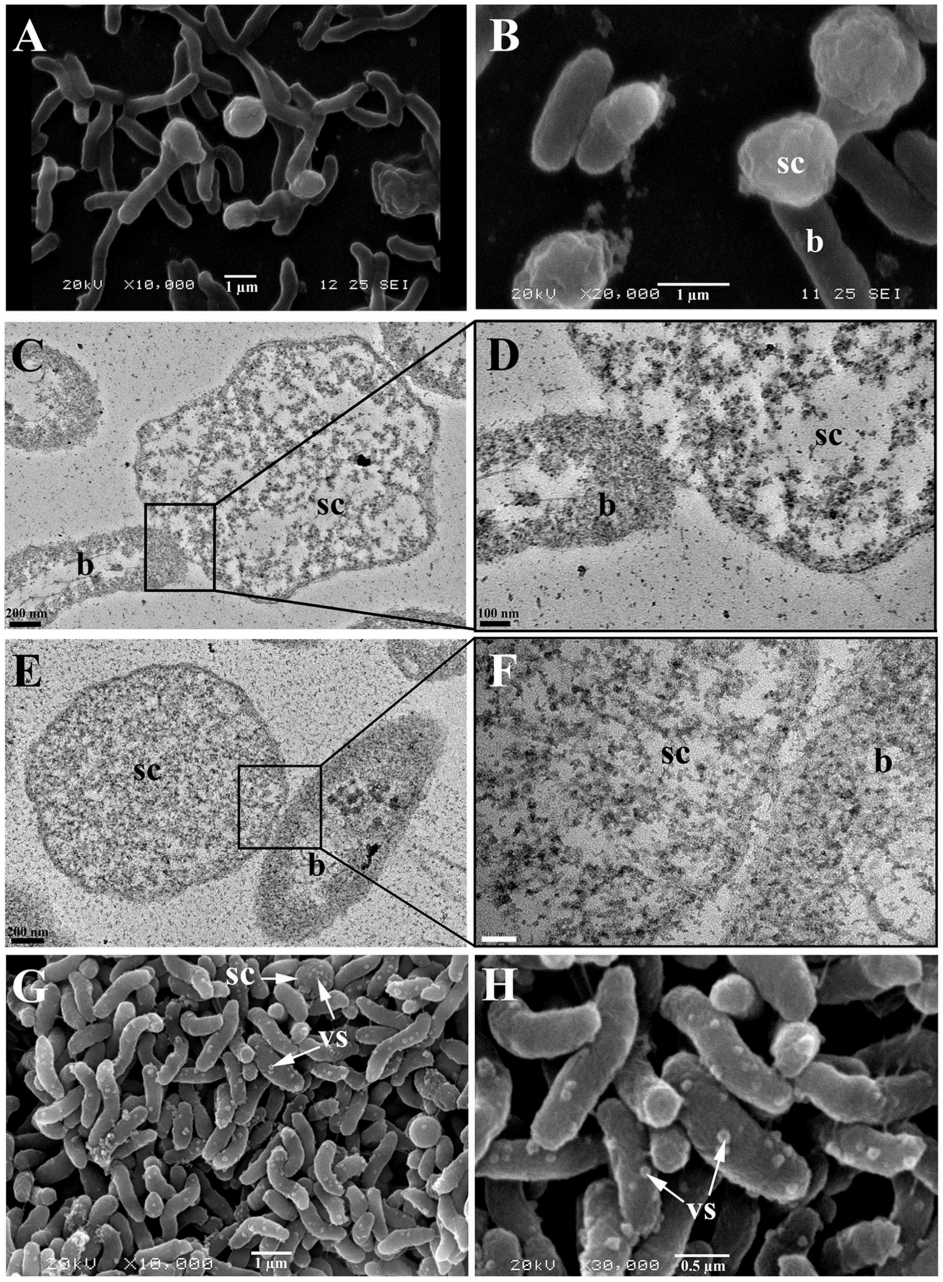
**Spherical components and bacterial cells from biofilms formed by ***V. tapetis*** observed by SEM (A,B,G,H) and TEM (C–F)**. **(D,F)** are enlargements of the squares of **(C,E)**, respectively. b, bacteria; sc, spherical components; vs, vesicles; vs and sc are shown with a write arrows **(G,H)**. Scale bars: 200 nm **(C,E)**; 100 nm **(D)**; 50 nm **(F)**.

The observation of the biofilm surface by SEM allowed us to estimate that these components had an average diameter of about 1–2 μm (Figures 6A,B). They were therefore too large to be membrane vesicles (diameters of 50 to 250 nm), which are produced by various Gram negative bacteria (Zhou et al., 1998; Mashburn-Warren et al., 2008). SEM images furthermore showed that the spherical components had a different surface aspect (rough aspect) than typical bacteria, and seemed physically linked to the cells, as if a cell part was differentiating (Figures 6A,B). In order to elucidate the spherical component ultrastructure, we used TEM to examine cells detaching from 48 h biofilms. Whereas the nucleoids of bacteria were clearly distinguishable from their cytoplasm (clear zone in the cell center), the spherical components contained a mix of clear and dark zones, but the clear zones were not particularly located at the center (Figures 6C,E). Furthermore, no internal compartment was visible. Each spherical component was found in the proximity of a bacterial cell and they seemed linked to each other by their membranes (Figures 6D,F).

**Figure 7:**
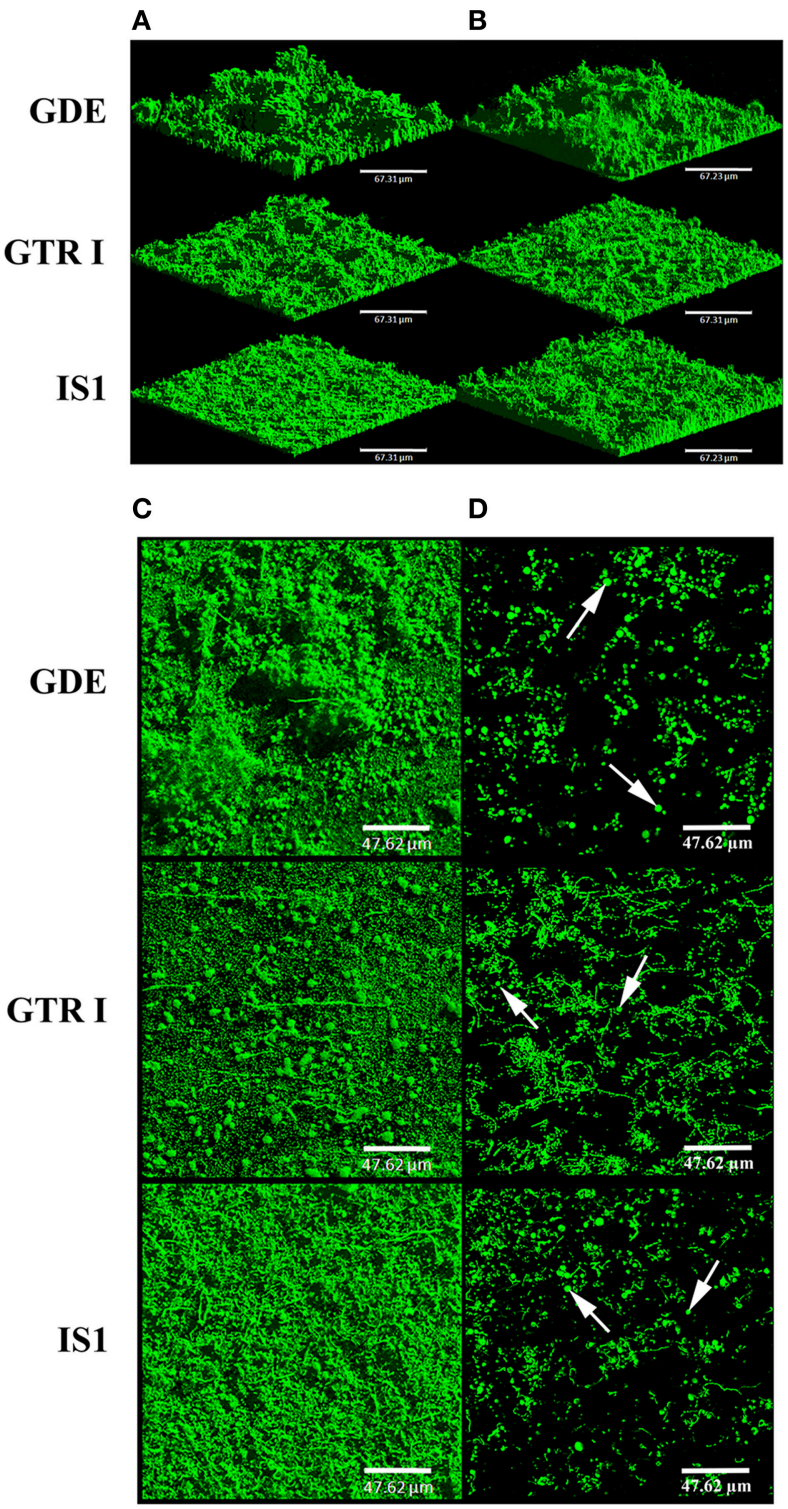
**Biofilms of ***V. tapetis*** GDE, GTR I, and IS1 strains**. The biofilms were grown for 24 h **(A)** and 48 h **(B–D)** and bacteria were stained with SYTO 9. 3D views **(A,B)** and top views **(C)** of biofilms were reconstructed from image stacks. To avoid the green background due to bacteria from the lower part of the biofilms, the upper part of each biofilm is shown with a single image from each stack in **(D)**. The white arrows indicate some spherical components. Scale bars: 67.31 μm **(A,B)**, 47.62 μm **(C,D)**.

In *V. tapetis* LP2 biofilms, we did not observe such spherical components using CLSM, but a few of them were visible by SEM at the biofilm surface (Figure 6G). Furthermore, we occasionally observed membrane vesicles both on bacteria and on spherical components of the LP2 strain (Figures 6G,H), illustrating the size difference between membrane vesicles and spherical components and indicating that the latter are of cellular nature. This scarcity of spherical components in LP2 biofilms prompted us to examine if the production of high amounts of spherical components is specific to the CECT4600 strain or if this property is shared by other *V. tapetis* strains. We therefore grew biofilms of the *V. tapetis* strains IS1, GDE, and GTR I, which are all pathogenic against clams of the *Veneridae* family: *R. philippinarum, D. exoleta*, and *T. rhomboides* (Table 1). All these strains displayed growth rates in liquid cultures which were similar to the CECT4600 one (Table 1). Spherical components were abundant at the surfaces of biofilms of these three strains (Figure 7). GDE was the most productive strain, its biofilm displaying aggregates of these spherical components, as seen on Figures 7C,D. GDE biofilms had a more heterogeneous surface (Figures 7A,B) than the reference strain CECT4600 (Figure 1) and achieved an average biovolume of 4.5 μm^3^.μm^−2^ after 48 h of growth (Figure 2). The GTR I strain formed biofilms which were less heterogeneous than the GDE ones (Figures 7A,B). GTR I biofilms harbored filamentous cells (Figures 7C,D) and numerous spherical components were visible at their surface (Figure 7D). The biovolume of GTR I biofilms did not exceed 2 μm^3^.μm^−2^ (Figure 2). The *R. philippinarum* pathogen IS1 strain formed homogeneous biofilms (Figures 7A,B) with an architecture very similar to the CECT4600 biofilm one (Figure 1) and an average biovolume of 8 μm^3^.μm^−2^ (Figure 2). IS1 biofilms also contained spherical components (Figures 7C,D), in a similar amount as CECT4600 biofilms. These spherical components are thus abundantly present in the biofilms of all the strains isolated from bivalves of the *Veneridae* family.

### Effect of culture conditions

**Figure 8:**
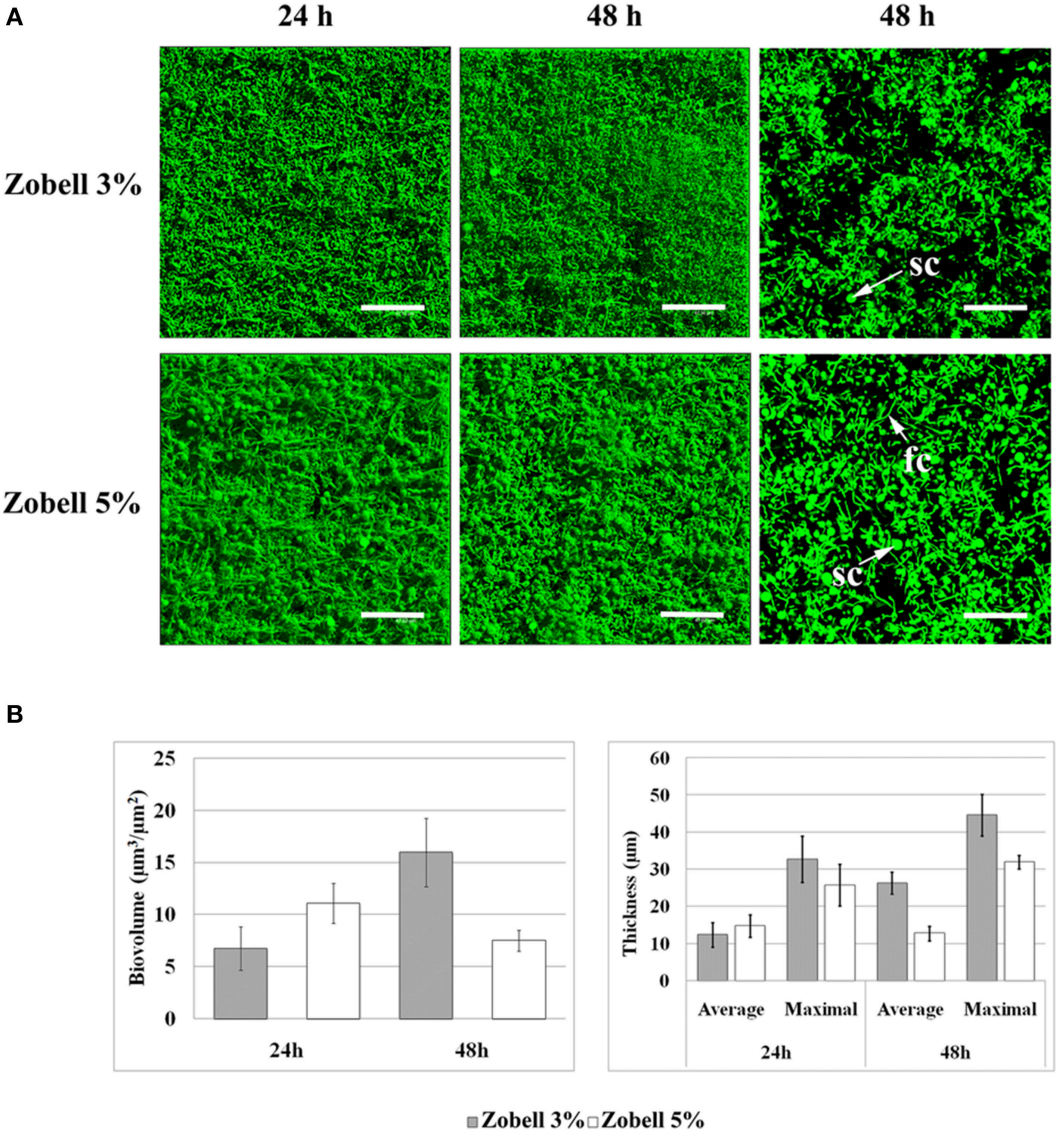
**Effect of increasing salinity on ***V. tapetis*** CECT4600 biofilm development**. **(A)**: CLSM images of 24 and 48 h biofilms formed by *V. tapetis* CECT4600 in Zobell 5%. Bacteria were stained with SYTO 9. Left and middle: top views of biofilms reconstructed from image stacks; right: single images from each stack (arrows: sc, spherical components; fc, filamentous cells). Scale bars: 47.62 μm. **(B)**: comparison of biovolume and thickness values of *V. tapetis* CECT4600 biofilms grown in Zobell 3% and in Zobell 5%. The values were determined by image analysis using COMSTAT software. Data are the means for at least three observations from three independent experiments (at least nine results), standard deviations are shown as a bar. All average biovolumes are significantly different (*p* < 0.001).

Temperature and salinity are subject to great variations in coastal environments where clams can be found. They are known to affect clam immune parameters and BRD expression, since a temperature of 21°C or a salinity of 4% led to lower BRD prevalence than a temperature of 14°C (Paillard et al., 2004) or a salinity of 2% (Reid et al., 2003). However, it is unknown if these parameters may also affect *V. tapetis* biofilm formation. To address this question, we chose the highest temperature (23°C) and the highest sea salt concentration [50 g.l^−1^ of sea salts in Zobell medium (Zobell 5%)] which did not affect the *V. tapetis* CECT4600 growth in liquid medium compared to our standard conditions (18°C and Zobell 3%; data not shown). At 23°C, we failed to observe any difference in biofilm formation by *V. tapetis* CECT4600 compared to 18°C during the first 24 h, but the biofilm biovolume did not keep increasing at 23°C between 24 and 48 h (data not shown). By contrast, when salinity was increased by using Zobell 5%, more spherical components were present in *V. tapetis* CECT4600 biofilms (Figure 8A). Furthermore, filamentous CECT4600 cells were abundant in biofilms grown in Zobell 5% (Figure 8A), which was not the case in Zobell 3% (Figure 1). This morphological change was not observed in liquid culture in Zobell 5% (data not shown). Salinity also affected the kinetics of biofilm biovolume: it was higher at 24 h of growth in Zobell 5% than in 3% (11 vs. 7 μm^3^.μm^−2^), but it decreased between 24 and 48 h down to 7 μm^3^.μm^−2^ in Zobell 5% whereas it increased up to 16 μm^3^.μm^−2^ during the same time frame in Zobell 3% (Figure 8B).

## Discussion

In this study, we characterized for the first time the biofilm formed by the clam pathogen *V. tapetis* CECT4600. We showed that *V. tapetis* CECT4600 is able to form biofilms *in vitro* on glass under dynamic conditions. After the initial attachment of planktonic bacteria to the surface, which could involve flagella and/or a swimming behavior, *V. tapetis* colonized the substratum by dividing without displaying surface motility, in contrast to other bacteria such as *Pseudomonas aeruginosa* (Barken et al., 2008). In *V. cholerae*, flagellar motility is also involved in early stage but repressed when biofilm is formed (Zhu et al., 2013). We suggested that it could be probably the same in *V. tapetis* since no motility was observed after initial attachment and no flagella was observed in SEM. *V. tapetis* CECT4600 and LP2 strains formed biofilms with notable differences in the distribution of matrix components and in the biofilm architecture. Interestingly, these two strains are very similar from a genetic point of view (Jensen et al., 2003), but they do not target the same type of organisms since LP2 is a fish pathogen.

Altogether, all the *V. tapetis* strains tested are able to form biofilms covering the entire glass surface in 24 h. At the surface of all *V. tapetis* biofilms, we observed unusual spherical components. They had an average diameter of about 1–2 μm, showed a different surface aspect than bacteria, contained DNA, and the presence of GFP in spherical components of *V. tapetis* CECT4600-GFP biofilms suggested that they contained proteins originating from *V. tapetis* cells. These observations and our SEM and TEM images support the hypothesis that the spherical components come from the bacterial cells to which they seem to remain attached. The occasional observation of membrane vesicles both on bacteria and on spherical components of the LP2 strain supports the idea that the spherical components are of cellular nature. TEM images showed that the spherical components were devoid of any kind of internal compartment. Furthermore, these images did not allow to clearly observe a central nucleoid, suggesting that the nucleoid might be more diffuse in spherical components than in bacterial cells. Berk et al. reported in 2012 that in *V. cholerae* biofilms, cells produced 50–200 nm diameter spheroids protruding away from the cell surface and containing matrix components of polysaccharidic nature (*Vibrio* polysaccharides, VPS; Berk et al., 2012). However, we failed to stain the *V. tapetis* spherical components with Calcofluor White (data not shown), which binds β1-3 and β1-4 polysaccharides, suggesting that those sugars are absent, which those not mean that there is no other exopolysaccharides. Coccoid cells were observed during the formation of viable but non-culturable (VBNC) *Vibrio cholerae* (Chaiyanan et al., 2007). However, VBNC cells are generally obtained upon environmental stresses such as exposure to low temperatures or nutrient deprivation for long periods (e.g., 30–60 days in Chaiyanan et al., 2007; Oliver, 2010). In our experiments, the biofilms were grown for only 48 h at the optimal growth temperature (18°C) under a flow of rich medium. The permanently renewed nutrients were available at least for bacteria of the upper biofilm part where the spherical components were observed. Even though we cannot formally ascertain that the spherical components are not VBNC cells, the culture conditions we used were unlikely to induce the development of the VBNC state. We failed to observe spherical components in all liquid cultures examined, which suggested that they might be biofilm-specific in our conditions. They were abundant at the surface of the biofilms of the four *V. tapetis* strains (CECT4600, IS1, GDE, GTR I) which are pathogenic toward clams of the *Veneridae* family (*R. philippinarum, D. exoleta*, and *T. rhomboïdes*), but very scarce at the surface of *V. tapetis* LP2 which is pathogenic toward the wrasse fish *Symphodus melops*. Unfortunately, no other *V. tapetis* strain non-pathogenic toward clams was available to confirm this trend. We thus cannot conclude on a possible relationship between the spherical components and the *V. tapetis* virulence toward clams. To our knowledge, this type of spherical components within a biofilm has not been previously reported in other bacterial species.

In marine invertebrates, disease prevalence is often controlled by environment factors (Harvell et al., 1999). Paillard et al. showed in 1997 the influence of temperature on BRD prevalence. *V. tapetis* growth is inhibited under 4°C and above 25°C; laboratory experiments have also demonstrated that a temperature of 21°C inhibits the development of BRD compared to 14°C and enhances the shell repair process (Paillard et al., 1997, 2004). In this study, we demonstrated that the *in vitro* biofilm formation by *V. tapetis* CECT4600 is not altered during the first 24 h by a growth at 23°C compared to 18°C. This indicates that elevated temperatures lead to a reduction of the BRD prevalence not by impairing *V. tapetis* biofilm development, but more likely by conferring to the clam better immune defenses to fight the disease agent, as described in 2004 by Paillard et al.

Environmental salinity has previously been correlated with disease incidence in Manila clam (Reid et al., 2003) and in several bivalve species (Chu et al., 1993; Hauton et al., 2000). Reid et al. reported in 2003 that the disease prevalence was significantly lower at a salinity of 4% than at 2%, which was consistent with an impairment of clam immune parameters at 2%. We showed here that *V. tapetis* CECT4600 is able to form biofilms at a high salinity (5%), but biovolumes and cells within the biofilm were affected. More spherical components were present at the biofilm surface in this condition, and numerous filamentous cells were observed. These changes were not observed in liquid culture. Filamentous bacterial morphology has already been described as potentially involved in bacterial survival in response to the sensing of environmental changes (Justice et al., 2008). Our observations on effects of culture parameters thus showed that *V. tapetis* is able to form biofilms in conditions in which the BRD prevalence is affected. It suggests that *V. tapetis* biofilm formation could play a role in the persistence of the pathogen in clam without inducing BRD symptoms. Biofilm development by *V. cholerae* facilitates its persistence in the environment and the host and plays a fundamental role in the epidemic cycles of this pathogen (Watnick and Kolter, 1999).

This first study describing the *V. tapetis* biofilm formation and characteristics yields the bases to decipher the role of biofilms in the pathogenic cycle of this bacterium toward clams. Future works will be undertaken to observe *V. tapetis* biofilm formation *in vivo*.

## Funding

SR is the recipient of a doctoral fellowship from Région Bretagne and was supported by the Axis 1 of GIS Europôle Mer. This work was performed in the framework of LabexMER “A Changing Ocean” ANR-10-LABX-19-01, which is a cluster of Excellence funded by the French “Investissements d'Avenir” program, supported by French Ministry of Research and Education.

### Conflict of interest statement

The authors declare that the research was conducted in the absence of any commercial or financial relationships that could be construed as a potential conflict of interest.

## Supplementary material

Supplemental Movie 1 *****V. tapetis*** CECT4600 biofilm formation (first 18 h)**. Time-lapse movie, an image was recorded every 10 min with a CLSM. 1 s of the movie is corresponding to ~52 min of experiment.

Supplemental Movie 2 *****V. tapetis*** LP2 biofilm formation (first 16 h)**. Time-lapse movie, an image was recorded every 10 min with a CLSM. 1 s of the movie is corresponding to ~52 min of experiment.
